# DSG2 expression is low in colon cancer and correlates with poor survival

**DOI:** 10.1186/s12876-020-01588-2

**Published:** 2021-01-06

**Authors:** Tingting Yang, Xuan Gu, Lizhou Jia, Jiaojiao Guo, Qi Tang, Jin Zhu, Wei Zhao, Zhenqing Feng

**Affiliations:** 1 Key Laboratory of Antibody Technique of National Health Commission, Nanjing Medical University, Nanjing, 211166 China; 2 Department of Pathology, Nanjing Medical University, Nanjing, 211166 China; 3 Department of Pathology, Nanjing First Hospital, Nanjing Medical University, Nanjing, 210006 China; 4 Huadong Medical Institute of Biotechniques, Nanjing, 210000 China; 5 Jiangsu Key Lab. of Cancer Biomarkers, Prevention and Treatment, Collaborative Innovation Center for Cancer Personalized Medicine, Nanjing Medical University, Nanjing, 211166 China

**Keywords:** DSG2, Colon cancer, Prognosis, Tissue microarray, Immunohistochemistry

## Abstract

**Background:**

Desmoglein2 (DSG2) is a transmembrane protein that helps regulate intercellular connections and contributes to desmosome assembly. Desmosome are associated with cell adhesion junctions, which play an important role in cancer progression specially cancer cell migration and invasion. However, DSG2 expression in colon cancer (CC) and its association with CC patients’ overall survival (OS) are still unclear.

**Methods:**

We collected 587 CC samples, 41 colitis tissues and 114 pericarcinomatous tissues, as well as corresponding clinicopathological data about the patients who contributed them. All samples were tested immunohistochemically in tissue microarrays. Kaplan–Meier method was used for calculating patient survival. Univariate and multivariate analyses was used for investigating DGS2 link with CC patient’s clinicopathological factors. Bioinformatics analysis was also used in study.

**Results:**

The results showed that DSG2 expression was lower in CC tissues than in pericarcinomatous tissues (*P* < 0.001). DSG2 expression was associated with differentiation (*P* = 0.033), lymph node metastasis (*P* = 0.045), distant metastasis (*P* = 0.006) and AJCC stage (*P* < 0.001). Univariate analysis indicated that poor OS in patients with CC was associated with low DSG2 expression (*P* < 0.001), tumor size (*P* < 0.001), lymph node metastasis (*P* < 0.001), distant metastasis (*P* < 0.001), AJCC stage (*P* < 0.001) and venous invasion (*P* < 0.001). In multivariate analysis, low DSG2 expression (*P* < 0.001), distant metastasis (*P* < 0.001), AJCC stage (*P* = 0.002), venous invasion (*P* < 0.001) were independent prognostic factors for CC patients. Bioinformatics analysis indicated that low DSG2 expression affects protein activation, regulates the P53-related pathway in CC, and activates the EGFR pathway.

**Conclusions:**

The results suggest that low DSG2 expression is associated with poor survival for CC patients. DSG2 could be a prognostic biomarker for CC.

## Background

Among malignant tumors, colon cancer (CC) is the third most common in both morbidity and mortality, with roughly 1.2 million new cases and 600,000 deaths worldwide every year [1]. Age is the strongest risk factor for CC; whereas its incidence rate is low in people younger than 50 years old, the number of Chinese patients with CC is expected to increase greatly in coming years as the median age of the Chinese population increases [2] Five-year survival rates for localized stage and regionally spread CC are 90.1% and 69.2%, respectively. However, the 5-year survival rate for distantly spread colon cancer is only 11.7% [3]. Early invasion or spread of CC indicates higher malignancy and poor prognosis. Surgery is the main treatment for both early- and late-stage CC, but tumors recur in 30%-50% of all cases, usually presenting as metastasis [4] Chemotherapy involves the use of anti-neoplastic agents that do not significantly distinguish between cancer and normal cells. Therefore, finding specific biomarkers for CC diagnosis and therapy is imperative [5, 6].

The desmoglein2 (DSG2) gene is located at chromosome 18q12.1. It encodes a single transmembrane protein. As a member of the classical cadherin family, DSG2 is calcium-dependent and assembles to make desmosomes. Ramani et al. found that shedding of soluble DSG2 was increased in pancreatic cancer, and that the loss of DSG2 may lead to cancer invasion [7]. Low DSG2 expression has been reported in aggressive prostate cancer [8], squamous lung cancer [9] and gastric cancer [10]. DSG2 can be used as a prognostic biomarker for cancer patients. Yang Liu et al. confirmed that DSG2 was decreased in KLF5 knockdown cells, and was related to intestinal barrier function. Dysfunction of intestinal barrier could lead to increasing intestinal permeability and intestinal inflammatory disease [11]. Patients with intestinal inflammatory diseases have higher risks of CC compared with the general population [12]. In cancer progress, inhibition of cell–cell adhesion is an indispensable first step in the metastatic process. However, few studies have investigated DSG2 expression and its function in CC.

To evaluate the role of DSG2 in CC prognosis and diagnosis, we assessed DSG2 expression in CC tissues and adjacent mucosa tissues by immunohistochemistry (IHC) staining in tissue microarrays (TMAs). The relationship between DSG2 expression and patients’ overall survival (OS) and clinicopathological factors were calculated by univariate and multivariate analysis. Bioinformatics methods were used to study DSG2-related signaling pathway.

## Methods

### Human tissue samples and homologous clinicopathological data

We collected 587 CC tissues, 41 colitis tissues and 114 pericarcinomatous tissues (total of 742 samples) from Nanjing First Hospital, Nanjing Medical University, which were collected from patients treated between 2006 and 2012. All samples were formalin-fixed and paraffin-embedded. Homologous clinicopathological data were also collected, including patients’ sex, age, tumor location, histological type, differentiation grade, tumor size, lymph node metastasis, distant metastasis, AJCC stage, venous or perineural invasion, preoperative CEA and CA199 serum level and Ki67 expression. Before surgical treatment, patients had no histories of radiotherapy or immunotherapy. Overall survival (OS) refers to the period from the initial biopsy confirming the diagnosis until death. CC staging was calculated according to the latest AJCC Cancer Staging Manual [13]. The research was approved by the hospital’s human research ethics committee. Written informed consent was obtained from the patients for scientific research of their tissue samples.

### TMA and IHC staining

All colon tissues samples (*diameter* = 1 mm) were cut by core tissue biopsy from normal paraffin blocks which were made from resected tissues. Section location for samples were selected by a pathologist. Every TMA block included 7 × 10 different tissues. A total of 10 colon TMAs was processed by a Tissue Microarray System (Quick Ray, UT06, UNITMA, Korea). Cut sections from paraffin blocks (4 µm) were placed on super frost-charged glass microscope slides. The slides were deparaffinized, rehydrated and incubated with 3% H_2_O_2_ to block endogenous peroxidase activity. Then sections were incubated in polyclonal rabbit anti-human DSG2 IgG overnight at 4℃ (dilution 1:300; 13684-T44, Sino biological, China) and washed three times by phosphate-buffered saline (PBS). The reaction was performed by EnVision method (Dako, Carpinteria, CA, USA). Sections were incubated with 3,3′-diaminobenzidine chromogen solution for 10 min, then counter stained with hematoxylin and covered by resin. PBS instead of DSG2 antibody was used as a negative control. The slices were evaluated by using Vectra 3.0 Automated Quantitative Pathology Imaging System (PerkinElmer, Connecticut, USA). The imaging system could automatically identify sample points. Staining intensity was defined as: 0 (no staining); 1+ (weak staining); 2+ (moderate staining); and 3+ (intense staining). The last DSG2 expression score of every sample was calculated by the staining intensity multiplied by the percentage of cell staining. The minimum score could be 0 (no staining) and maximum score could be 300 (3 × 100%). The final scores were recorded for analysis.

### GBA analysis and gene set enrichment

Guilt-by-association (GBA) analysis was used to investigate DSG2 function. Pearson’s correlated protein-coded genes were analyzed for relationships with DSG2. Gene set enrichment analysis (GSEA v2.2.2 software http://www.broadinstitute.org/gsea) was used to identify the role of DSG2 in CC samples. The Kyoto Encyclopedia of Genes and Genomes (KEGG) was used for DSG2 pathway function annotation. The Bioconductor Cluster Profiler Package was used for KEGG functional enrichment analysis and was connected to GGplot2 for visualization. Protein–protein interaction (PPI) was constructed by Cytoscape_v3.7.0. The network of DSG2 and associated proteins was analyzed by STRING v10.5 (https: //string-db.org).

### Statistical analysis

All statistical analyses were performed by SPSS 18.0 statistical software package (SPSS Inc. Chicago, IL). The scores from TMAs were divided into two parts by cutoff value using the X-tile software program (The Rimm Lab at Yale University; http://www.tissuearray.org/rimmlab) [14]. Two groups of data were tested by Student’s *t* test and χ^2^ test. Patient survival was calculated by the Kaplan–Meier method. Univariate and multivariate analyses used the Cox proportional hazards regression model. *P* < 0.05 was regarded as significant.

## Results

### DSG2 protein expression in CC by immunohistochemistry staining

**Fig. 1:**
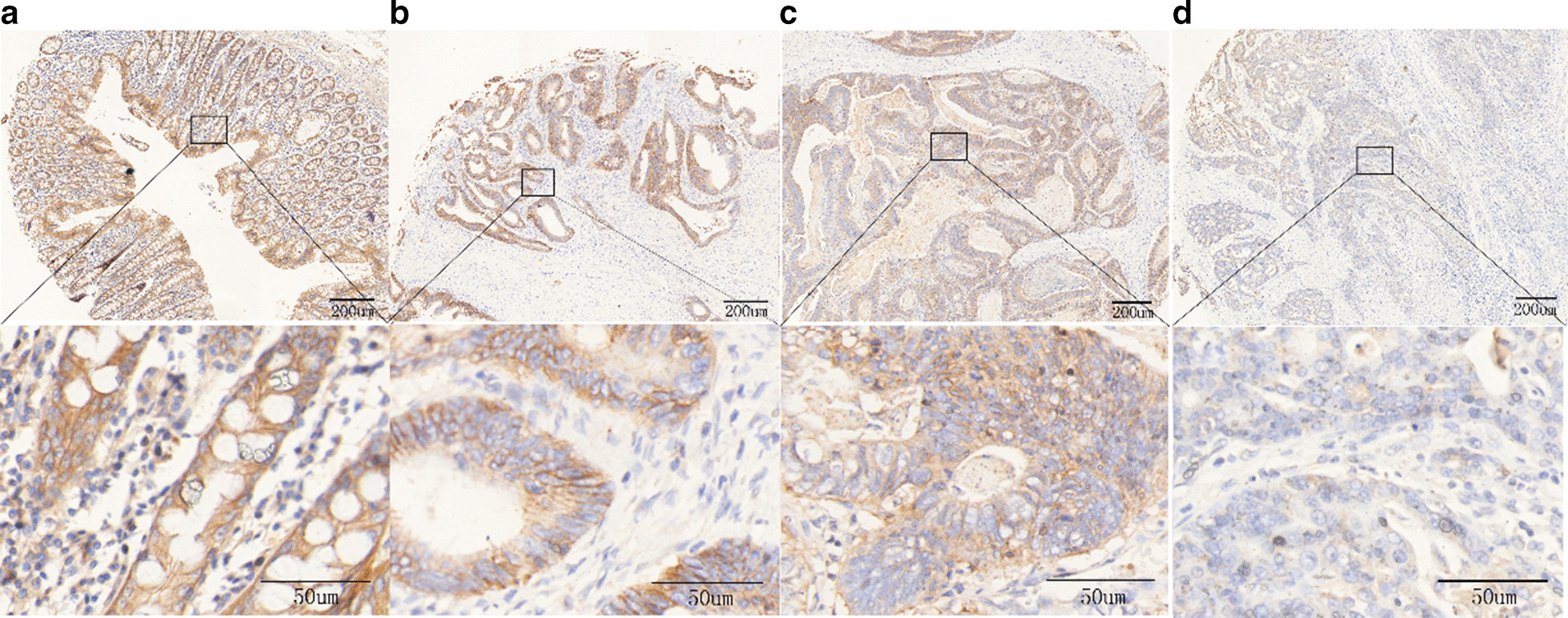
DSG2 protein expression in colon tissues by immunohistochemistry staining. **a** Colitis tissue with high DSG2 expression, **b** high-level intraepithelial neoplasia of colon tissue with high DSG2 expression, **c** low-level intraepithelial neoplasia of colon tissue with low DSG2 expression, **d** adenocarcinoma with low DSG2 expression. Top row is the representative images of the DSG2 expression in colon tissues. Bottom row is the magnified images of the part area in the top row

DSG2 protein expression was detected by IHC in CC tissues and related pericarcinomatous tissues and colitis tissues. DSG2 was expressed mainly on cells’ membranes and partly in the cytoplasm. TMAs’ scores were divided into groups with low or no expression and high expression using a cutoff value, 130. The results showed that CC tissues had low DSG2 expression, compared with that pericarcinomatous tissues (Fig. 1). Low DSG2 protein expression was observed in 56.22% (330/587) of CC samples, but only in 29.27% (12/41) colitis and 28.95% (33/114) of pericarcinomatous tissues (Table 1).

**Table 1:** Dsg2 expression in colon tissues

Characteristics	n	Dsg2 expression (%)		X^2^	P
		Low or no	High		
Colitis	41	12 (29.27%)	29 (70.73%)	36.26	< 0.001
Carcinoma	587	330 (56.22%)	257 (43.78%)		
Pericarcinomatous tissue	114	33 (28.95%)	81 (71.05%)		

Dsg2 protein expression incolitis, carcinoma and pericarcinomatous tissue were detected by immunohistochemical staining

### Correlations of DSG2 protein expression and clinicopathological factors in CC

The relationship between DSG2 expression level and CC patients’ clinicopathological factors was analyzed, using IHC-stained TMA. DSG2 expression was significantly associated with differentiation (χ^2^ = 8.77, *P* = 0.033), lymph node metastasis (χ^2^ = 6.21, *P* = 0.045), distant metastasis (χ^2^ = 7.90, *P* = 0.005) and AJCC stage (χ^2^ = 18.54, *P* < 0.001). However, DSG2 expression was not associated with the other factors, including age and Ki67 (Table 2).

**Table 2:** Dsg2 expression level and CC patients’ clinicopathological characteristics

Characteristics	n	Dsg2		Pearson X^2^	P-value
		Low and no	High		
Total	587	330 (56.22%)	257 (43.78%)		
Gender				0.54	0.463
Male	351	193 (54.99%)	158 (45.01%)		
Female	236	137 (58.05%)	99 (41.95%)		
Age				0.79	0.376
< 60	215	126 (58.60%)	89 (41.40%)		
≥ 60	372	204 (54.84%)	168 (45.16%)		
Location				3.02	0.389
Right	188	110 (58.51%)	78 (41.49%)		
Transverse	77	46 (59.74%)	31 (40.26%)		
Left	114	67 (58.77%)	47 (41.23%)		
Sigmoid	208	107 (51.44%)	101 (48.56%)		
Histological type				0.42	0.515
Adenocarcinoma	522	291 (55.75%)	231 (44.25%)		
Mucinous/SRCC^a^	65	39 (60.00%)	26 (40.00%)		
Differentiation				8.77	0.033*
Well	156	83 (53.21%)	73 (46.95%)		
Moderate	314	173 (55.10%)	141 (44.91%)		
Poor	99	58 (58.59%)	41 (41.41%)		
Others^b^	18	16 (88.89%)	2 (11.11%)		
Tumor size				1.44	0.696
T1	42	25 (59.52%)	17 (40.48%)		
T2	61	37 (60.66%)	24 (39.34%)		
T3	200	115 (57.50%)	85 (42.50%)		
T4	284	153 (53.87%)	131 (46.13%)		
Lymph node metastasis				6.21	0.045*
N0	328	171 (52.13%)	157 (47.87%)		
N1	168	99 (58.93%)	69 (41.07%)		
N2	91	60 (65.93%)	31 (34.07%)		
Distant metastasis				7.90	0.005*
M0	495	266 (53.74%)	229 (46.26%)		
M1	92	64 (69.57%)	28 (30.43%)		
AJCC stage				18.54	< 0.001*
I	63	28 (44.44%)	35 (55.56%)		
II	199	95 (47.74%)	104 (52.26%)		
III	233	143 (61.37%)	90 (38.63%)		
IV	92	64 (69.57%)	28 (30.43%)		
Venous invasion				0.15	0.697
Negative	501	280 (55.89%)	221 (44.11%)		
Positive	86	50 (58.14%)	36 (41.86%)		
Perineural invasion				0.24	0.623
Negative	507	283 (55.82%)	224 (44.18%)		
Positive	80	47 (58.75%)	33 (41.25%)		
Preoperative CEA, ng/ml				1.17	0.557
≤ 5	259	150 (57.92%)	109 (42.08%)		
> 5	285	154 (54.04%)	131 (45.96%)		
Unknown	43	26 (60.47%)	17 (39.53%)		
Preoperative CA199, ng/ml				1.06	0.588
≤ 37	261	151 (57.85%)	110 (42.15%)		
> 37	281	152 (54.09%)	129 (45.91%)		
Unknown	45	27 (60.00%)	18 (40.00%)		
Ki67				0.58	0.444
Negative	181	106 (58.56%)	75 (41.44%)		
Positive	406	224 (55.17%)	182 (44.83%)		

*P < 0.05 indicated a significant associated with clinical characteristics

^a^Mucinous carcinoma is 57 cases; SRCC (signet-ring cell carcinoma) is 8 cases

^b^No clear differentiation stage diagnosis

### DSG2 protein expression was associated with poor prognosis in CC

**Fig. 2:**
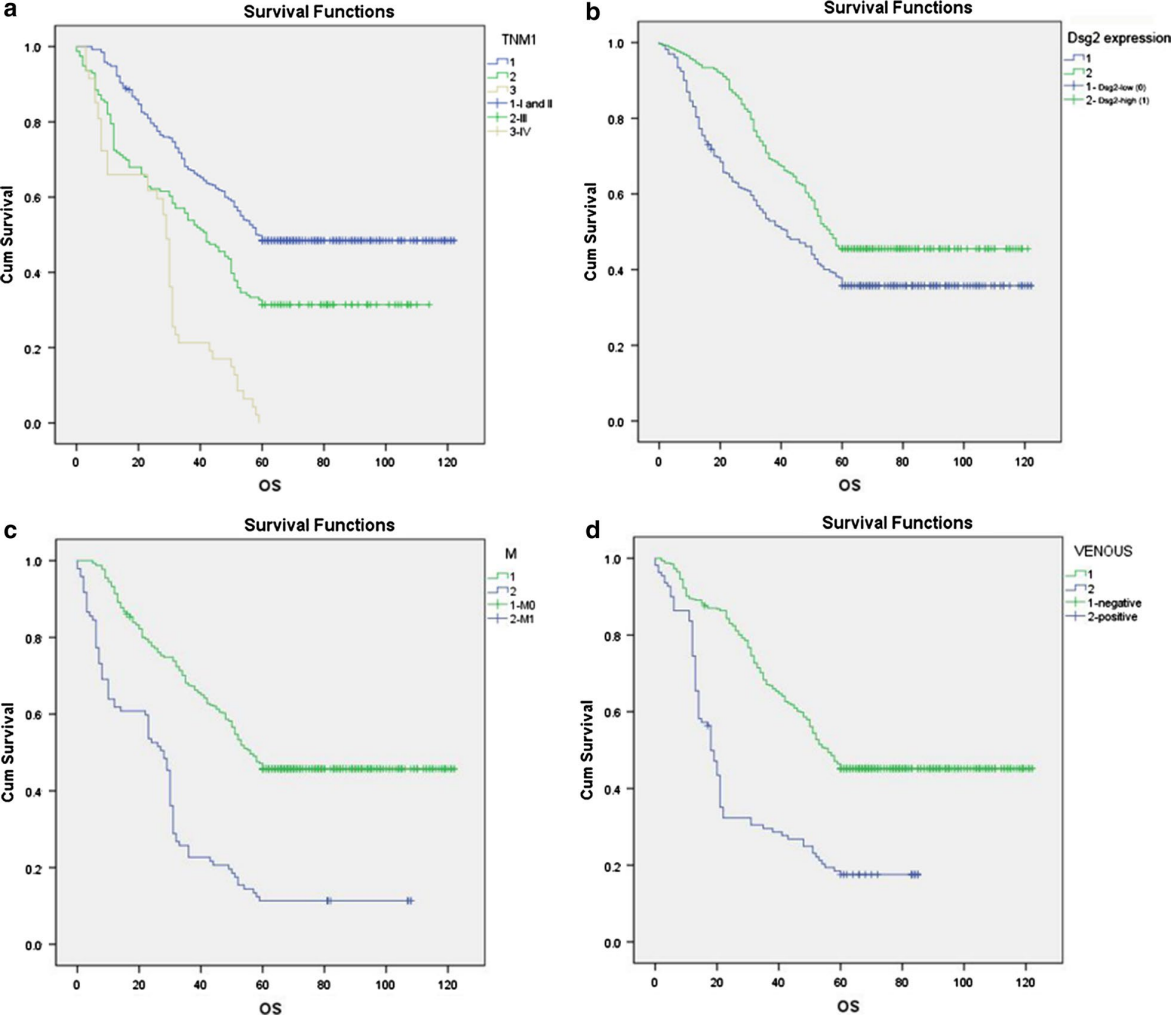
Survival curve of CC patients by Kaplan–Meier plots and log-rank test. **a** The overall survival curve of patients with TNM (I and II) was significant higher than TNM(III and IV). **b** The overall survival curve of patients with DSG2 high expression was significantly higher than DSG2 low expression. **c** The overall survival curve of patients with distant metastasis was significantly lower than without distant metastasis. **d** The overall survival curve of patients with venous invasion was significantly lower than without venous invasion. All *P* value in log-rank test is under 0.001

Univariate and multivariate analyses were used to evaluate the prognosis value of DSG2 expression and other clinical factors among CC patients. Univariate analysis showed that the CC patients’ OS was associated with DSG2 expression (HR = 0.996, *P* < 0.001), tumor size (HR = 1.295, *P* < 0.001), lymph node metastasis (HR = 0.642, *P* < 0.001), distant metastasis (HR = 3.098, *P* < 0.001), AJCC stage (HR = 1.331, *P* < 0.001) and venous invasion (HR = 2.735, *P* < 0.001). When the above significant factors were processed by multivariate analysis, low DSG2 expression (HR = 0.996, *P* < 0.001), distant metastasis (HR = 3.245, *P* < 0.001), AJCC stage (HR = 1.254, *P* = 0.002) and venous invasion (HR = 2.815, P < 0.001) were independent factors for OS (Table 3). Kaplan–Meier survival curves visually displayed the associations between TNM stage III + IV, low DSG2 expression, distant metastasis, and venous invasion, and CC patients’ OS (Fig. 2).

**Table 3:** Univariate and multivariable analysis of prognostic factors for overall survival in CC

	Univariate analysis		Multivariate analysis		P-value	95%CI
	HR	P-value	95% CI	HR		
Dsg2 expression						
Low or no	Reference					
High	0.996	< 0.001*	0.995–0.998	0.996	< 0.001*	0.995–0.998
Age(year)						
≤ 60	Reference					
> 60	1.042	0.708	0.841–1.290			
Gender						
Male	Reference					
Female	1.119	0.102	0.992–1.149			
Location						
Right/Transverse	Reference					
Left/Sigmoid	1.004	0.919	0.922–1.094			
Histological type						
Adenocarcinoma	Reference					
Mucinous/SRCC^a^	0.963	0.790	0.731–1.269			
Differentiation						
Well/Moderate	Reference					
Poor/Others^b^	1.202	0.056	0.947–1.380			
Tumor size						
T1/T2	Reference					
T3/T4	1.295	< 0.001*	1.144–1.465	1.164	0.078	0.917–1.332
Lymph node metastasis						
N0/N1	Reference					
N2	0.642	< 0.001*	0.549–0.750	0.931	0.081	0.846–1.633
Distant metastasis						
M0	Reference					
M1	3.098	< 0.001*	2.421–3.965	3.245	< 0.001*	2.462–4.276
AJCC stage						
I/II	Reference					
III/IV	1.331	< 0.001*	1.186–1.495	1.254	0.002*	1.09–1.442
Venous invasion						
Negative	Reference					
Positive	2.735	< 0.001*	2.145–3.486	2.815	< 0.001*	2.175–3.644
Perineural invasion						
Negative	Reference					
Positive	1.481	0.075	0.951–1.730			
CEA(ng/ml)						
≤ 5	Reference					
> 5	1.031	0.781	0.833–1.275			
CA199(ng/ml)						
≤ 37	Reference					
> 37	0.950	0.634	0.768–1.175			
Ki67						
Negative	Reference					
Positive	0.995	0.965	0.805–1.230			

Dsg2 expression, tumor size, lymph node metastasis, Distant metastasis, AJCC stage and venous invasion were included for multivariate analysis

*P < 0.05, statistically significant

^a^Mucinous carcinoma is 57 cases; SRCC (signet-ring cell carcinoma) is 8 cases

^b^No clear differentiation stage record

*HR* hazard ratio, *CI* confidence interval

### DSG2 expression in CC was tested by Gene set enrichment analysis (GSEA) and Pearson correlation analysis

**Fig. 3:**
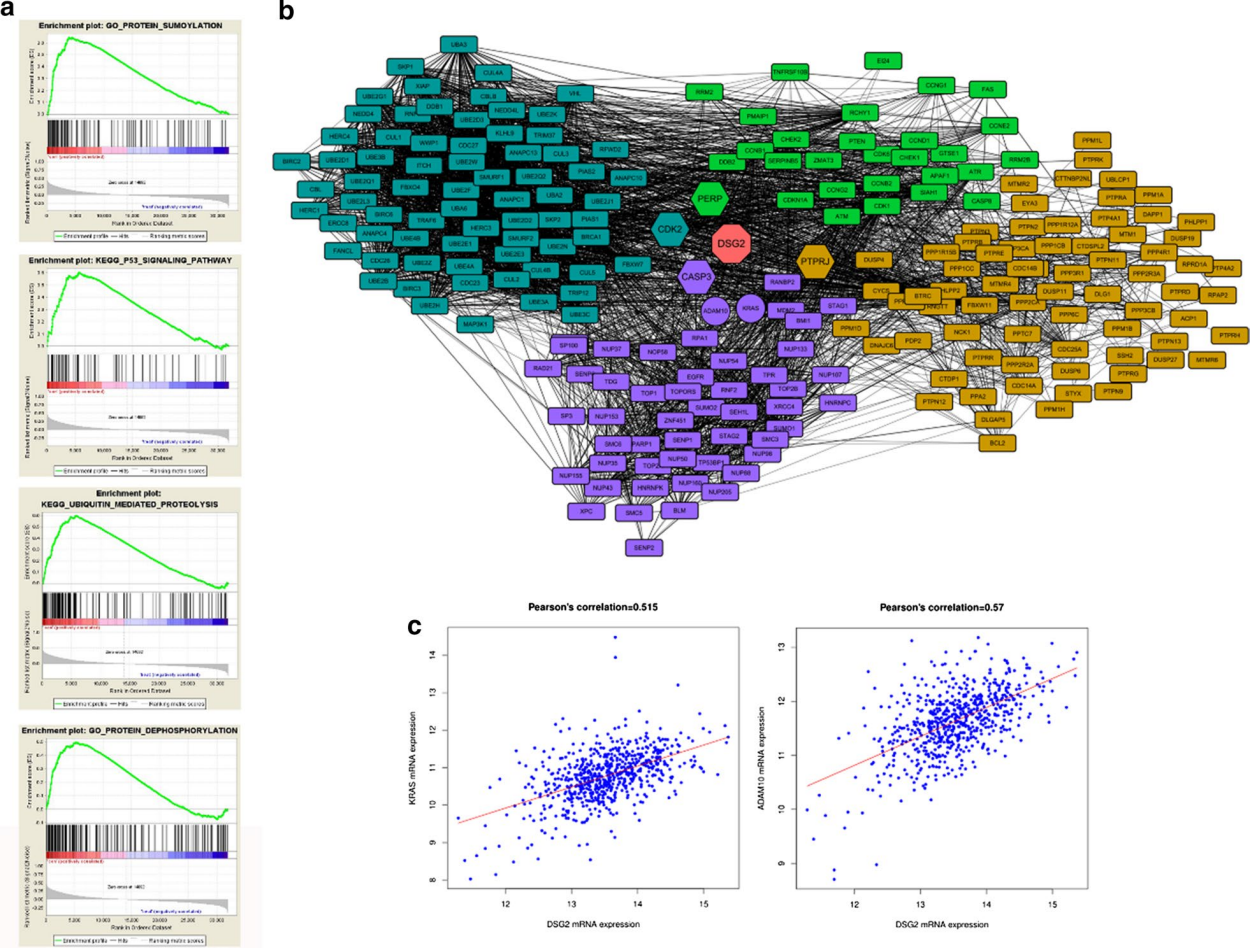
The role of DSG2 in related signalling pathways? of colon cancer. **a** These four pathways were figured out be significant associated with DSG2 low expression. protein sumoylation; P53 signaling pathway; ubiquitin mediated proteolysis and protein dephosphorylation. **b** Protein–protein interactions were extracted by STRINGv10.5 database and built in cytoscape_v3.7.0. proteins which were from same pathway were showed in same color. **c** KRAS and ADAM10 were positive correlated with DSG2 expression in colon cancer samples

To ascertain DSG2′s role in colon tumor formation and progression, an integrative analysis of CC microarray expression profiles was performed from GEO datasets. TCGA-colorectal cancer was used as basic dataset. The low DSG2 expression group was seen to be obviously enriched for protein sumoylation, P53 signaling pathway, ubiquitin-mediated proteolysis and protein dephosphorylation (Fig. 3a, Table 4). The protein network confirmed that KRAS and ADAM10 expression were associated with DSG2 (Fig. 3c). The protein network associated proteins in above four pathways were associated with DSG2 protein (Fig. 3b). Four different colors represented different proteins coded by genes that participated in the above four pathways. ADAM10 and KRAS were linked with DSG2 protein in the network.

**Table 4:** Four enriched pathways for differential expression Dsg2 in CC

Pathway	ES	NES	FDR p-value	Normal p-value	FWER p-value
Protein_sumoylation	0.64	2.22	0.005	< 0.001	0.025
P53_signaling_pathway	0.61	2.15	0.003	< 0.001	0.003
Ubiquitin_mediated_proteolysis	0.60	2.26	< 0.001	< 0.001	< 0.001
Protein_dephosphorylation	0.50	2.17	0.008	< 0.001	0.052

*ES* enrichment score, *NES* normal enrichment score, *FDR* false discovery rate, *FWER* family wise error rate

## Discussion

DSG2 was first found in colon tissues, and is also known as human desmoglein colon (HDGC) [15]. As a transmembrane protein, DSG2 is connect with desmocollin on the cell membrane and regulates cell adhesion. Desmoglein has four subtypes, of while DSG2 is the most widely expressed subtype in tissues [16]. Funakoshi et al. confirmed that desmosome junctions contribute to the maintenance of normal intestinal epithelial columnar morphology and antagonize epithelial-to-mesenchymal transition (EMT) which is a classical phenomenon for the metastasis of CC cells in humans [17, 18].

Our research indicated that DSG2 was low expression in CC, which are associated with CC’s TNM stage, differentiation and AJCC stage. Kaplan–Meier analysis and the Cox proportion hazards model analysis showed that patients with low DSG2 expression have a poorer prognosis compared with high DSG2 expression.. Univariate and multivariate analyses suggested that DSG2 was an independent factor for CC patients’ OS. All findings indicate that DSG2 could be a prognostic biomarker for CC.

Kundu et al. reported that reduction of desmosome proteins could decrease cell adhesion and promote metastasis in CC [19]. Vishnu C Ramani’s study showed that DSG2 expression was reduced in pancreatic tumors, and it suggested that loss of desmosomal proteins play an important role in pancreatic cancer invasion [7]. Various classes of cell adhesion molecules, including desmosomes, are affected in cancers and their expression and function are regulated via various mechanisms [20]. The components that comprise intercellular junctions, such as desmosome play a critical role in cell adhesive function. The expression level of DSG2 which form the integral part of desmosomal adhesive core were also affected in tumors [21]. DSG2 was low expression in gastric cancer [22], squamous cell carcinoma [23], lung cancer [9] and prostate cancer [24]. Our results also indicated that low DSG2 expression was associated with CC TNM stage and patients’ OS. According to the aforementioned studies, low expression of DSG2—the key protein in desmosome assemble—may induced tumor adhesion function disorder as well as tumor cell invasion and migration. Conversely, the research of Kamekura R et al. suggested that desmosome proteins can promote cell proliferation in CC [25]. The study showed that DSG2 and DSC2 play opposite roles in tumor proliferation. Desmosome have a complex junction construction that is assembled by multiple proteins [26]. Altered expression of desmosome proteins may promote cancer development in certain contexts, but DSG2 affects carcinogenesis in different tumors in different ways, such as tumor composition, protein expression level, subcellular localization and tissue-specific proteins [27]. Further, these different findings may be due to the limitations of these carcinogenic alternative models. In physiological mouse models with intact immune systems in which cancers develop in the appropriate tissue microenvironment, the results indicate that the downregulation of desmosomes may contribute to malignant progression [28]. In our research, CC tissues collected from patients can better reflect the true condition of the tumor.

Our bioinformatics analysis indicated that P53 was associated with abnormally expressed DSG2 in CC. PMP-22 (PERK) is a tetraspan membrane protein that is transcriptionally activated by the P53 tumor suppressor [29]. And PERK is located to desmosomes in stratified epithelia and is crucial for proper desmosome assembly [30]. The link between those two proteins provides us more direction in DSG2 mechanism research. all three metabolic pathways (protein sumoylation, ubiquitin-mediated proteolysis and protein dephosphorylation) have the close relationships with DSG2 expression in CC based on RNA expression level. From normal epithelial cells, mutated cells, into immortalized tumor cells, cellular metabolic activity gradually increases abnormally. Upstream of the three metabolic pathways were biochemistry changes that affect cell mitosis and proliferation. Our bioinformatics network also showed that all four pathways were linked with DSG2 protein. These results suggest that DSG2 is related to tumor suppression and cancer-related protein activation. Pearson analysis said that ADAM10 and KRAS were positively linked with DSG2 at the mRNA level. The study of Klessner, et al. found that DSG2 shedding occurred in tumor cells, and ADAM10 shed N- cadherin and E-cadherin from the cell surface and reduced adhesion of neuronal and epithelial cells [31]. ADAM10 knock down resulted in greater accumulation of DSG2 fragments. ADAM10 was regulated by EGFR expression. When EGFR was blocked by PKI, ADAM10 could not be activated [32]. EGFR is a therapeutic target in several human tumors, including CC, for which the anti-EGFR target antibody, panitumumab, is administered as monotherapy. KRAS encodes a small GTP-binding protein that acts as a self-inactivating signal transducer by cycling from GDP-bound states in response to stimulation by EGFR [33]. Mutant KRAS in many tumors is associated with lack of response to EGFR inhibitors. Therefore, DSG2 abnormal expression may participated in activating the EGFR-related pathway in CC malignant progression.

DSG2 was expressed mainly on cells’ membranes and partly in the cytoplasm and was low expression in CC compared with pericarcinomatous tissues and colitis tissues. DSG2 was associated with some clinicopathological factors in CC including differentiation, lymph node metastasis, distant metastasis and AJCC stage. DSG2 was associated with poor prognosis in CC**.** DSG2 could be an independent prognostic biomarker for CC.

## Conclusions

DSG2 is low expressed in CC compared with pericarcinomatous tissues and is an independent factor for CC patient’s OS. Results indicated that DSG2 could be a prognostic biomarker for CC.

## Data Availability

The datasets used and analyzed during the current study available from the corresponding author on reasonable request.

## Abbreviations

CC: colon cancer
DSG2: desmoglein2
OS: overall survival
AJCC: American Joint Commission on Cancer
TMAs: tissue microarrays
IHC: immunohistochemistry
PBS: phosphate-buffered saline
GSEA: gene set enrichment analysis
KEGG: Kyoto Encyclopedia of Genes and Genomes
PPI: protein–protein interaction
EMT: epithelial-to-mesenchymal transition
